# Temperature and pathogen exposure act independently to drive host phenotypic trajectories

**DOI:** 10.1098/rsbl.2021.0072

**Published:** 2021-06-16

**Authors:** Tobias E. Hector, Carla M. Sgrò, Matthew D. Hall

**Affiliations:** ^1^ School of Biological Sciences, Monash University, Melbourne, VIC 3800, Australia; ^2^ Centre for Geometric Biology, Monash University, Melbourne, VIC 3800, Australia

**Keywords:** temperature, stress, pathogen, phenotypic trajectory, infectious disease, life-history

## Abstract

Natural populations are experiencing an increase in the occurrence of both thermal stress and disease outbreaks. How these two common stressors interact to determine host phenotypic shifts will be important for population persistence, yet a myriad of different traits and pathways are a target of both stressors, making generalizable predictions difficult to obtain. Here, using the host *Daphnia magna* and its bacterial pathogen *Pasteuria ramosa*, we tested how temperature and pathogen exposure interact to drive shifts in multivariate host phenotypes. We found that these two stressors acted mostly independently to shape host phenotypic trajectories, with temperature driving a faster pace of life by favouring early development and increased intrinsic population growth rates, while pathogen exposure impacted reproductive potential through reductions in lifetime fecundity. Studies focussed on extreme thermal stress are increasingly showing how pathogen exposure can severely hamper the thermal tolerance of a host. However, our results suggest that under milder thermal stress, and in terms of life-history traits, increases in temperature might not exacerbate the impact of pathogen exposure on host performance, and vice versa.

## 1. Introduction

As a result of global change, populations are experiencing an increase in the occurrence of thermal stress [1,2]. Simultaneously, increasing temperatures are driving a shift in the distribution of pathogens and the severity of infectious disease outbreaks [3–7]. How individual's response to global change will increasingly be determined by the interactions between thermal stress and pathogen exposure [8–10]. For example, infection has been shown to severely hamper a host's thermal tolerance, suggesting individuals exposed to disease will be particularly sensitive to extreme thermal stress such as heat waves [11–13]. Increases in temperature and pathogen exposure, however, will not only shape host thermal tolerance, but will also drive changes in all aspects of a host's phenotype, including development, growth, fecundity and population-level processes (e.g. [9,14–17]). Population persistence will therefore be shaped by how temperature change and pathogen exposure directly interact to shift multiple facets of a host's phenotype, in addition to the complex role that temperate plays in driving pathogen transmission strategies [18–20] and host–pathogen interactions more broadly [21,22].

Several outcomes are possible when an individual experiences both a pathogen and elevated temperatures. These stressors can act additively, where the impact they have on an individual is simply the sum of their independent effects. Alternatively, antagonistic and synergistic interactions can occur, where the cumulative impact of two stressors is less than, or more than, the sum of their independent effects, respectively [23–25]. An antagonistic interaction between thermal stress and pathogen exposure suggests that disease outcomes under thermal change might be less severe than expected, while any synergistic interaction would instead place a host at greater risk than expected. An understanding of how co-occurring stressors interact to determine shifts in host performance is therefore vital if we are to understand the fitness consequences of thermal change and pathogen exposure acting in concert.

Studies of the impacts of temperature and pathogen exposure often focus on their effects on individual host traits (e.g. [14–17]). However, organisms are comprised of multiple correlated traits that together form the integrated phenotype [26]. While determining how stressors impact individual traits gives insight into how they may affect specific components of individual or population performance, a univariate approach can be misleading if the magnitude or direction of any phenotypic shifts depend on which trait is being assessed or on the correlations between traits [27]. By taking a multivariate view, it is possible to understand phenotypic shifts in response to environmental change in a more holistic manner [27–29]. Indeed a phenotypic shift can manifest via changes in the magnitude and/or the direction of phenotypic change in multivariate space—collectively known as the phenotypic trajectory [30,31]. By assessing the multivariate phenotypic trajectory of a host, we can more fully understand whether the phenotypic impact of a pathogen will be enhanced or lessened under rising temperatures.

In this study, we tested how exposure of *Daphnia magna* to a bacterial pathogen interacts with increased temperatures to drive shifts in a host's phenotype. Across 11 life-history traits, we used phenotypic trajectory analysis to compare the magnitude and direction of temperature-induced phenotypic changes for animals exposed or unexposed to a pathogen. As part of this process, we assessed the independent impacts of elevated temperature during the maternal generation and on the focal generation to dissect trans-generational from direct thermal effects, but found maternal temperature effects to be relatively minor. Traits studied included pace of life traits such as developmental timing and intrinsic population growth rates, in addition to fecundity traits, somatic growth and lifespan. Using this integrated multivariate approach, we assessed whether temperature increases might exacerbate or lessen the impact of pathogens on host performance.

## 2. Methods

*Daphnia magna* Straus is a freshwater cyclically parthenogenic crustacean from the northern hemisphere [32]. *Pasteuria ramosa* is a natural Gram-positive bacterial pathogen of *Daphnia* that infects during filter feeding before entering the body and reproducing, causing reduced fecundity and lifespan of its host [33–35]. Here, we used *Daphnia* genotype BE-OMZ-M10 infected with one of three *P. ramosa* genotypes (C1, C14, C20), which vary in their rates of within-host proliferation as well the virulence they cause to their host [34,36]. Prior to the experiment female *Daphnia* were raised individually for three generations in 70 ml jars filled with 50 ml of artificial *Daphnia* Medium (ADaM; [37]) and maintained under standard conditions (see electronic supplementary material).

### (a) Temperature treatments and infection

On their day of birth, maternal generation females were taken from clutches 3–5 of the standardized animals and placed at either 20°C or 25°C (maternal temperature treatment). Experimental individuals were then collected from clutches 3–5 of the acclimated mothers on their day of birth and placed at either 20°C or 25°C (focal temperature treatment) in a fully factorial design. The first temperature represents maternal and developmental acclimation (*Daphnia* experience early development within the brood pouch of their mothers) and the second temperature represents direct thermal acclimation on the focal individuals from birth, which lasted over the rest of their lives, including during growth, maturity and the infection period.

A total of 496 females were set up individually in the experimental generation in a fully factorial design, with 31 individuals per treatment (2 maternal temperatures × 2 focal temperatures × [3 pathogens + uninfected controls]). For the infection treatments, individual *Daphnia* was exposed to 40 000 *P. ramosa* spores from one of the three pathogen genotypes over 2 days (20 000 per day) starting 3 days after birth. The infection took place over 3 days in 70 ml jars filled with 20 ml ADaM, after which all animals were transferred to 50 ml of fresh ADaM and maintained at their respective temperatures.

### (b) Life-history traits

All individuals were monitored daily from birth until death and various life-history traits were recorded: age and body size at maturity, body size at day 20, age at and size of the first clutch, lifetime fecundity, total number of clutches, mean clutch size and lifespan. Body size measures the length of *Daphnia* from the top of the head above the eye to the base of the tail spine. Two additional traits were also calculated, including, somatic growth rate (Gj) and individual intrinsic rate of increase (*r*), calculated by solving the Euler–Lokta equation (see electronic supplementary material).

### (c) Statistical analysis

All analyses were conducted in R (v. 3.6.2; R Development Core Team). Figures were produced using *ggplot2* [38] and *cowplot* [39].

Owing to the relatively minor influence of maternal temperature across most traits, we controlled for any effect of maternal temperature (two levels: 20°C or 25°C) by including it as an additive term and focussed instead on the interaction between focal temperature (two levels: 20°C or 25°C) and infection treatment (four levels: control, C1, C14 or C20). Lifetime fecundity and total number of clutches were both log-transformed and intrinsic rate of increase (*r*) was squared before all analyses. For the univariate analyses, age at maturity and age at first clutch were analysed using a generalized linear model with Poisson distributed errors. All other traits were analysed using linear models. The significance of fixed effects was then tested using analysis of variance (Type III, white corrected to account for residual heterogeneity; *car* package [40]).

Prior to multivariate analysis, all traits were standardized to have a mean of zero and a standard deviation of one. A permutation-based MANOVA was used to analyse how temperature and pathogen exposure influence multivariate host phenotypes using the fixed effect structure described above [41,42]. We then used phenotypic trajectory analysis to calculate the relative magnitude (*D*) and angle (*θ*) of any shift in host phenotype (phenotypic trajectory) across temperature for each infection treatment [30,31,41] and visualized these patterns using principal component analysis (PCA) on all 11 traits across the two treatments. All multivariate analyses were conducted using the *RRPP* package [42]. See the electronic supplementary material for details on the sample sizes for each treatment combination.

## 3. Results

**Figure 1.**
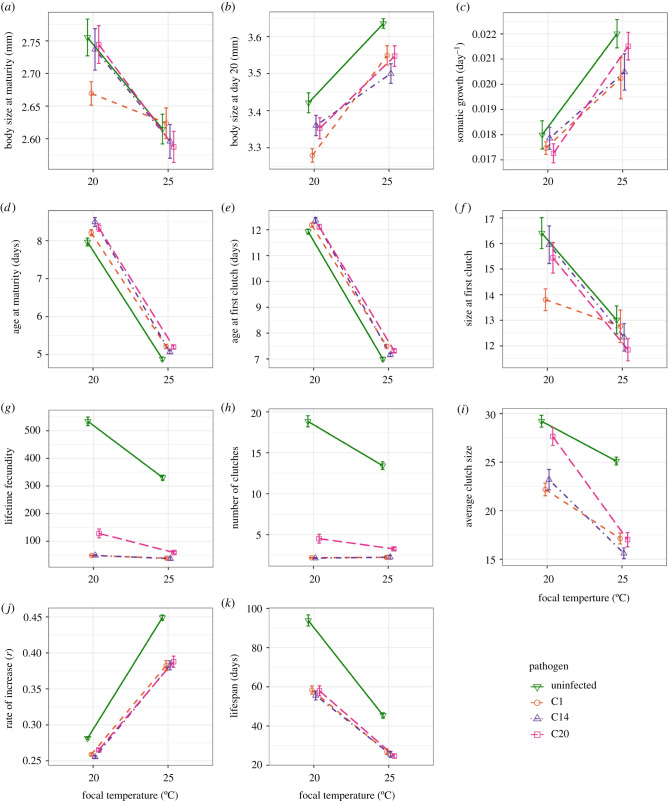
Univariate responses to infection treatment and focal temperature, showing the mean (±s.e.) for each treatment group. Due to generally small effects of maternal temperature treatment on most variables, for visual clarity data were pooled across maternal temperature. See electronic supplementary material, figure S1 for data split across maternal temperature.

We observed three distinct outcomes for the effects of temperature and pathogen exposure on individual traits (figure 1; electronic supplementary material, table S1 for ANOVA results). First, for traits like body size and age at maturity, and size and age of the first clutch, trait values decreased as temperature increased, with patterns dominated by the effect of temperature alone. Second, for somatic growth rate and body size at day 20, trait values increased with temperature, with temperature again being the dominant driver of variation, but with pathogen exposure also leading to a reduction in trait values. For all other traits, any response depended on an interaction between temperature and pathogen exposure. With lifetime fecundity, the total number of clutches and lifespan, trait values deceased at warmer temperatures, but much more so for uninfected animals. By contrast, the intrinsic rate of increase increased at higher temperatures, where the detrimental impact of pathogen exposure was also greatest. Average clutch size was lowest at warmer temperatures, with the greatest reduction seen in infected individuals.

**Figure 2.**
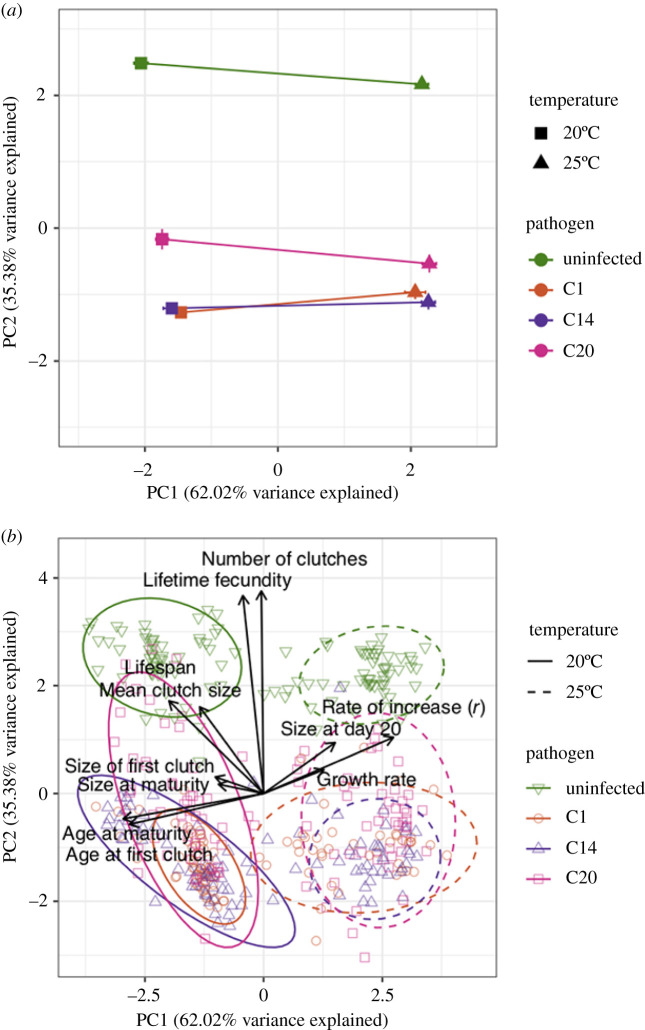
Principal component plots showing: (*a*) the phenotypic trajectories in response to temperature and pathogen exposure across 11 life-history traits (±s.e.) and (*b*) PCA plots showing all observations grouped by temperature and infection treatment. Length of arrows represent the relative magnitude of the contribution each trait made towards PC1 and PC2, while their orientation represents the direction of any shift, and relative direction shows trait covariation. Ellipses represent 95% confidence bands.

The MANOVA and phenotypic trajectory analysis results reveal that, after accounting for correlations among trait responses, temperature and pathogen exposure act largely independently to drive host phenotypic trajectories (electronic supplementary material, table S2; table 1 and figure 2*a*). The magnitude of the multivariate response to temperature (*D* in table 1) was consistent across most exposure treatments indicating that the strength of temperature-driven phenotypic change is not altered by infection (figure 2*a*). The direction of each trajectory in multi-trait space was also relatively aligned (*θ* in table 1 and figure 2*a*), with any difference in angles between two trajectories all less than 20°, indicative of a very mild convergence of the phenotypic trajectories for each exposure treatment at warmer temperatures (figure 2*a*; electronic supplementary material, table S2 for the MANOVA results).

**Table 1.** Phenotypic trajectory analysis comparing the difference in magnitude (*D*) and angle (*θ*) of phenotypic shifts caused by temperature across infection treatments based on multivariate changes in 11 life-history traits. Significant *p*-values are in italics.

treatment comparisons	magnitude difference (*D*)	*Z*	*p*-value	angle difference (*θ*)	*z*	*p*-value
uninfected–C1	0.703	4.206	<*0.001*	17.201	3.217	*0.006*
uninfected–C14	0.386	1.621	0.077	18.784	3.519	*0.004*
uninfected–C20	0.211	0.285	0.349	18.185	3.548	*0.006*
C1–C14	0.318	1.055	0.163	17.997	3.089	*0.008*
C1–C20	0.492	2.434	*0.027*	19.266	3.64	*0.002*
C14–C20	0.174	−0.039	0.437	11.702	1.127	0.134

Visualization of the traits contributing to the phenotypic trajectories revealed that temperature and infection shape phenotypic trajectories in very different ways (figure 2*b*). Temperature primarily shifted phenotypes across ‘pace of life’ traits, including developmental timing traits and population growth rates. By contrast, pathogen exposure impacted reproductive potential, causing large reductions in both lifetime fecundity traits and lifespan.

## 4. Discussion

Rising temperatures have the potential to either reduce or exacerbate the impact of infection on a host's phenotype, depending on whether the interaction between these two stressors is antagonistic or synergistic [23,24]. Our results, however, suggest that changes in host life-history traits, at least, may not be subject to such complex interactions. A phenotypic trajectory analysis of 11 different life-history traits in *Daphnia* showed that trait changes driven by temperature were not substantially modified by infection (table 1 and figure 2). Temperature generally influenced ‘pace-of-life’ traits, with warmer temperatures accelerating rate-based traits, such as age at maturity and population growth rate (*r*), potentially reflecting the intrinsic link between temperature and metabolic rate (see also [28,43]). Pathogen exposure, by contrast, shaped traits related to the reproductive potential and longevity of its hosts. This may be expected in this system, as the pathogen *P. ramosa* severely reduces the fecundity and lifespan of its host [15,16,34].

The lack of a synergistic interaction, whereby phenotypic differences between infected and uninfected individuals are increased at warmer temperatures, suggests that the fitness costs of infection for a host might not be universally worse under warmer temperatures [4,5,8]. Instead, mild thermal stress and pathogen exposure appear to have largely independent and predictable effects on the phenotypic trajectory of a host. Thus, while pathogen exposure is known to make individuals far more sensitive to extreme thermal stress [11,12], we show the reverse is not necessarily true—elevated temperature may not always exacerbate the damage pathogens cause. We stress that this does not mean that the severity of an infectious disease outbreak for a host population will remain unchanged under warmer temperatures (e.g. [44]). Rising temperatures will also alter the likelihood a host becomes infected and the transmission dynamics for a pathogen [7,16,18,19,45], and changes in the reproductive capacity of a host, as documented here, will inevitably feed back into the supply of susceptible hosts on which the spread of infectious diseases depend [46].

But what about other host–pathogen systems? Here comparisons remain difficult as rarely are a full suite of host life-history traits, or host population growth rates, measured simultaneously under infection and thermal stress. Instead the focus is often on pathogen transmission and rates of increase [9]. In a similar *Daphnia*–fungus system, for example, infection reduced lifespan, but the relative reduction was reasonably consistent across temperatures [19], consistent with our broad results. But equally, studies in this same system have found that both shifts to cooler temperatures and warmer temperatures can lead to a smaller relative reduction in fecundity or lifespan of infected individuals [17,47]. These results reinforce how only a simultaneous assessment of multiple life-history traits offers a complete picture of the way environmental change will shift host phenotypes [27–29].

In conclusion, the joint effects of thermal stress and pathogen exposure are increasingly being recognized as a key determinant of population persistence for natural populations [8,9,48]. Using a multivariate approach, we have shown that these two stressors may act independently, and that pathogen exposure may not necessarily exacerbate the impacts of global change on host life-history traits. We believe these results, even on a single host genotype, can be generalizable. Our previous work [11,12], and that of others [45], has shown similarities in the way different host genotypes respond to infection and thermal stress (at least in the direction of any response). We also observed that the magnitude and angle of phenotypic shifts due to temperature were remarkably consistent across three pathogen genotypes of varying transmission and virulence [34,36]. Nonetheless, assessing how temperature and pathogens interact to shape host phenotypic trajectories across a broad range of host–pathogen systems, populations and genotypes will help refine the conditions under which an increase in temperature does not exacerbate the impact of pathogen exposure on host performance (see [49]).

## Data accessibility

Data and code from this manuscript can be found via the Figshare repository at https://dx.doi.org/10.26180/13721266 [50].
